# Machine Learning with Alpha Toxin Phenotype to Predict Clinical Outcome in Patients with *Staphylococcus aureus* Bloodstream Infection

**DOI:** 10.3390/toxins15070417

**Published:** 2023-06-27

**Authors:** Brent Beadell, Surya Nehra, Elizabeth Gusenov, Holly Huse, Annie Wong-Beringer

**Affiliations:** 1 Alfred E. Mann School of Pharmacy and Pharmaceutical Sciences, University of Southern California, Los Angeles, CA 90089, USA; beadell@usc.edu (B.B.); gusenov@usc.edu (E.G.); 2 Viterbi School of Engineering, University of Southern California, Los Angeles, CA 90089, USA; nehra@usc.edu; 3 Department of Microbiology, Harbor-UCLA Medical Center, Torrance, CA 90502, USA; hhuse@dhs.lacounty.gov; 4 Department of Pharmacy, Huntington Hospital, Pasadena, CA 91105, USA

**Keywords:** *Staphylococcus aureus*, bacteremia, machine learning, platelets, α-toxin, thrombocytopenia

## Abstract

*Staphylococcus aureus* bloodstream (SAB) infection remains a leading cause of sepsis-related mortality. Yet, current treatment does not account for variable virulence traits that mediate host dysregulated immune response, such as SA α-toxin (Hla)-mediated thrombocytopenia. Here, we applied machine learning (ML) to bacterial growth images combined with platelet count data to predict patient outcomes. We profiled Hla phenotypes of SA isolates collected from patients with bacteremia by taking smartphone images of beta-hemolytic growth on sheep blood agar (SBA). Electronic medical records were reviewed to extract relevant laboratory and clinical data. A convolutional neural network was applied to process the plate image data for input along with day 1 patient platelet count to generate ML-based models that predict thrombocytopenia on day 4 and mortality. A total of 229 patients infected with SA strains exhibiting varying zone sizes of beta-hemolysis on SBA were included. A total of 539 images of bacterial growth on SBA were generated as inputs for model development. One-third of patients developed thrombocytopenia at onset, with an overall mortality rate of 18.8%. The models developed from the ML algorithm showed strong performance (AUC 0.92) for predicting thrombocytopenia on day 4 of infection and modest performance (AUC 0.711) for mortality. Our findings support further development and validation of a proof-of-concept ML application in digital microbiology, with a measure of bacterial virulence factor production that carries prognostic significance and can help guide treatment selection.

## 1. Introduction

*Staphylococcus aureus* (SA) is a leading cause of sepsis with mortality rates up to 40% [1]. A dysregulated host immune response during systemic infection to staphylococcal virulence contributes to poor outcomes [2,3]. Our laboratory previously showed that one in three patients with SA bacteremia (SAB) develops persistent growth of bacteria in the blood despite treatment with antibiotics, which significantly increases the risk of death [4]. An increased appreciation for a dysregulated host immune response as a driver of patient outcomes underscores the importance of incorporating measures of bacterial virulence factor production with prognostic significance to the diagnostic workup; in turn, these results may guide clinical decision-making.

SA utilizes an arsenal of virulence factors to subvert the host immune system. In particular, SA produces α-toxin (Hla), which is an oligomeric pore-forming hemolysin that binds to A Disintegrin and Metalloproteinase 10 (ADAM10), a membrane-spanning protease widely expressed on different cell types throughout the human body. Numerous studies have shown Hla to be a key driver of outcome during SA infection [5,6,7,8,9,10]. Nearly all SA strains encode Hla, but our group has previously shown highly variable expression of Hla among clinical strains that cause bacteremia [11]. Hla targets platelets, which are vital for orchestrating immune responses to systemic SA infection, to cause aberrant aggregation as well as premature platelet aging and depletion [6,12,13]. Importantly, patients with bloodstream infection due to high Hla-producing strains are at significant risk for developing thrombocytopenia and death [11]. Further, Hla production has been previously shown to be inhibited by select antistaphylococcal agents in the class of protein synthesis inhibitors (e.g., tedizolid, clindamycin) [14]. Additionally, two clinical trials in Phase 3 clinical development of a human monoclonal antibody targeting Hla by Aridis Pharmaceuticals are ongoing to investigate its use for the adjunctive treatment (NCT03816956) or prevention of ventilator-associated pneumonia due to SA (NCT05331885). 

Together, knowledge of the virulence potential of the infecting strain and patient-specific immune response are requisite information that enables precision infectious disease therapeutics. However, current management relies solely on the genus and species identification of the infecting strain and antimicrobial susceptibility profile without taking into consideration bacterial virulence factor production and host immune response. In the research setting, Hla production can be measured by the ability of SA isolates to cause rabbit red blood cell lysis (hemolytic activity), but assay complexity limits its feasibility for use in the clinical setting. Notably, Hla-producing SA strains exhibit beta-hemolysis when grown on sheep blood agar, and different beta-hemolytic zone sizes may provide a measurable qualitative readout of SA Hla production based on image interpretation [15].

Recent developments in artificial intelligence have led to an increased interest in applying data-driven machine learning (ML) to clinical decision-making in healthcare [16,17,18]. ML applications in the realm of digital microbiology have garnered much interest in aiding in diagnosis and treatment [19,20,21]. Distinct beta-hemolytic growth patterns of Hla-producing SA lends itself to ML-assisted digital microbiology. Here, we sought to develop a proof-of-concept machine learning model through training on plate images of bacterial growth and associated patient outcomes. In turn, the ML-based model could potentially be incorporated into the current workflow in clinical microbiology laboratories for reporting to the clinician the virulence potential of the infecting SA strain that carries prognostic significance.

## 2. Results

### 2.1. Bacterial Isolates and Patient Characteristics

**Figure 1:**
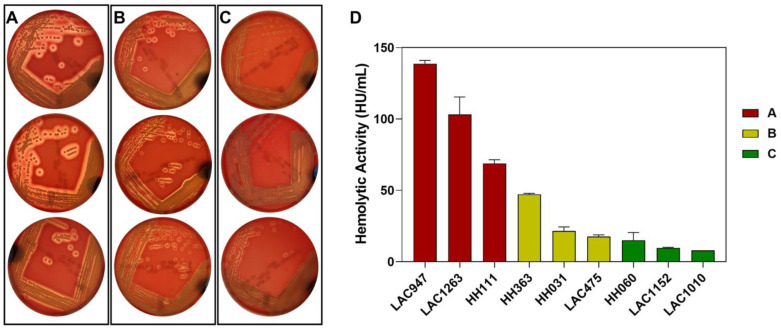
Representative plate images of beta-hemolytic growth taken by a smartphone as inputs to ML algorithm development. Nine representative images of clinical isolates grown on SBA plates are arbitrarily grouped into (**A**–**C**) hemolytic phenotypes based on the different zone size of hemolysis shown here with (**D**) corresponding degree of hemolytic activity in hemolytic units (HU/mL) as measured by rabbit erythrocyte hemolysis assay performed on bacterial supernatants. Error bars represent s.d.

A total of 229 patients with *S. aureus* bacteremia were included in the study, chosen based on growth of SA strains on SBA with visible zones of beta-hemolysis and completeness of relevant laboratory and clinical data. At least one-third of patients (34%) experienced thrombocytopenia (TC) on day 1 while 41% experienced TC on day 4 of bacteremia. Study patients were grouped by platelet count on day 1 of positive blood culture (NP, normal platelet count; TC, thrombocytopenia) and compared on comorbidities, infection source risk category, methicillin resistance of the infecting strain, severity of presentation, use of concurrent antiplatelet therapies, bacteremia duration, and mortality. (Table 1) The most common overall comorbidities were hypertension and diabetes, affecting nearly half of the patients in all subgroups. Patients with TC were more likely to be infected with high-risk source (34.6% vs. 15.9%, *p =* 0.002), have severe sepsis (75.6% vs. 41.7%, *p* < 0.0001) and septic shock (25.6% vs. 9.9%, *p =* 0.003), experience persistent bacteremia (55.1% vs. 31.8%, *p =* 0.001), and die (33.3% vs. 11.3%, *p =* 0.0001) when compared to those without TC. Slightly over one-third (36%) of the infecting isolates were MRSA. Study strains exhibited varying zone sizes of beta-hemolysis when grown on SBA (Figure 1), with corresponding hemolytic activity that ranged from 0 to 191.2 HU/mL (mean 29.9 HU/mL) (Supplementary Table S1). Six strains had null hemolytic activity but displayed very minor hemolytic zones on SBA plates.

### 2.2. Development of ML Models 

**Figure 2:**
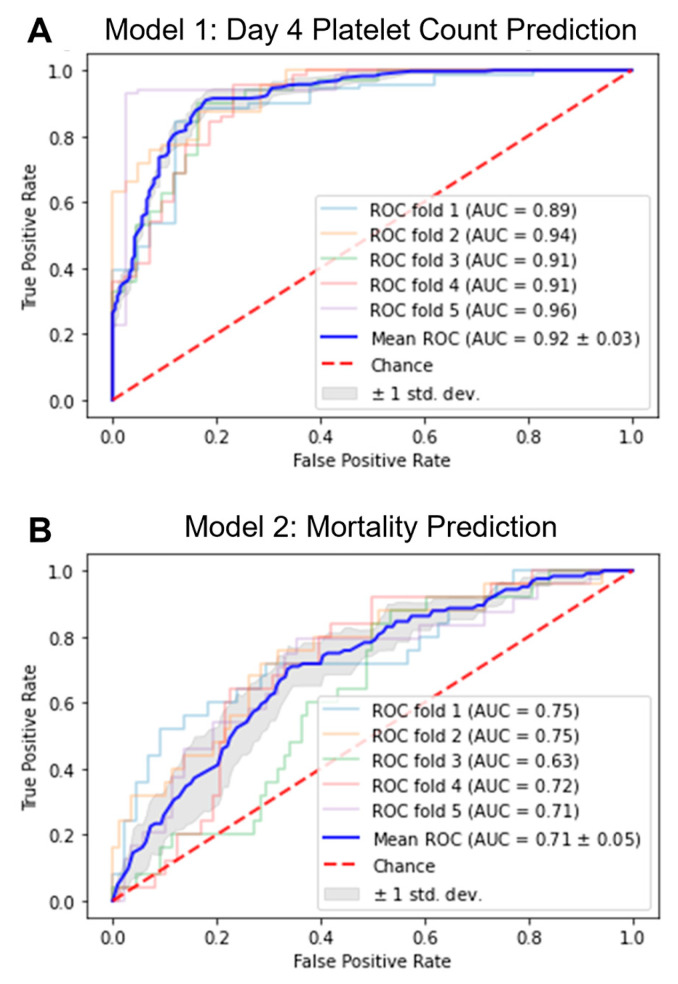
Receiver operating characteristic (ROC) curves for prediction of thrombocytopenia (**A**: Model 1) and mortality (**B**: Model 2) from 5 cross-validation experiments and the mean ROC. ROC curves from machine learning models 1 and 2 to predict thrombocytopenia on day 4 of bloodstream infection and mortality, respectively. Images of hemolytic growth pattern and patient platelet count on day 1 were used as inputs. Individual experiment ROC curves are displayed as transparent lines, and mean ROC across 5 separate experiments is illustrated by the solid blue line, with 1 standard deviation shown in grey. Chance (AUC = 0.5) is displayed as a dotted red line. Neural network architectures employed are ResNet50 (**A**) and InceptionV3 (**B**), respectively.

We have previously shown that patients who experienced TC on day 1 of bloodstream infection may recover by day 4 while others who had normal platelet counts on day 1 may develop TC on day 4 depending on the degree of Hla production of the infecting strain and that TC on day 4 was significantly associated with mortality [23]. A total of 539 images taken with a smartphone representing growth of 229 unique isolates on SBA were employed. We applied an ML approach to develop two models to predict: (1) thrombocytopenia (platelet count < 150 × 10^9^ L) on day 4 of infection and (2) 30-day mortality based on SBA plate images and patient platelet count on day 1 (Figure 2A,B). Performance metrics for the two ML models in predicting day 4 platelet count and 30-day mortality are described in Table 2. Overall model performance of the ML algorithms as indicated by the AUC value showed strong performance for prediction of TC on day 4 (AUC 0.920) and modest performance for mortality prediction (AUC 0.711) (Figure 2A,B).

### 2.3. Application of ML Models

**Figure 3:**
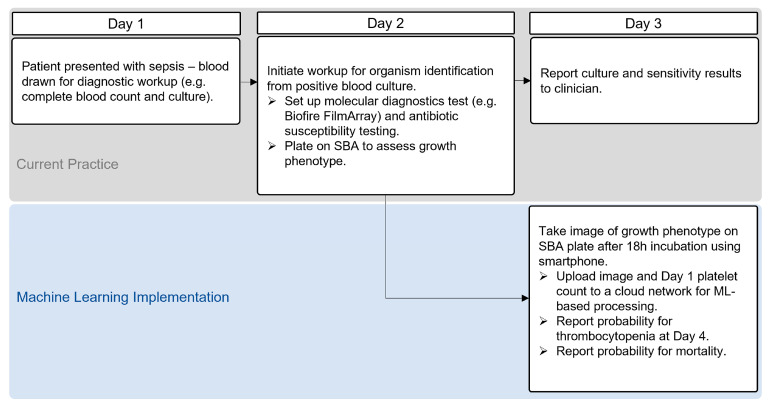
Proposed clinical microbiology workflow with implementation of machine learning applications. An image of the beta-hemolytic growth on sheep blood agar (SBA) plate would be taken on Day 3 for upload along with Day 1 platelet count into a cloud network for processing using an ML algorithm. An immediate output predicting Day 4 patient platelet status would be generated. Similarly, mortality risk may be predicted following the same process using the plate image and Day 1 platelet count as inputs.

The ML models developed are intended to demonstrate the proof-of-concept for clinical microbiologists to provide clinicians with measures of virulence factor production that carry prognostic significance. In the real-world scenario, we envision that suspected SA cultures from blood culture bottles would be directly sub-cultured onto SBA. As part of the existing workflow, the lab technologist would take a photograph image of SA growth on SBA plates using a smartphone and upload the image along with day 1 platelet count to a cloud network for processing by the ML algorithm. Expected readouts would include (1) risk probability for the patient developing thrombocytopenia early during the course of infection (day 4) and (2) probability of death. (Figure 3) Depending on the risk probability predicted and input from the antimicrobial stewardship team, the clinician would be able to individualize treatment by considering host–microbe interaction in addition to the traditional approach based primarily on organism susceptibility. The precision therapeutics approach may include the use of an antistaphylococcal agent we have previously shown to inhibit Hla (e.g., tedizolid) or an antistaphylococcal agent in combination with a platelet protective agent against Hla-mediated effects (e.g., ticagrelor) [14,24].

## 3. Discussion

Virulence-mediated dysregulation of the host immune response likely contributes to the high mortality associated with SA bloodstream infection. At present, treatment selection for SAB relies solely on isolate identification, antimicrobial susceptibility testing results, and drug safety without consideration for bacterial virulence and host immune response. We sought to apply an ML approach to measure SA virulence (Hla production) to predict risk of dysregulated host response (day 4 thrombocytopenia) and 30-day mortality. We demonstrated a proof-of-concept clinically relevant model that could be readily implemented into the existing clinical microbiology workflow with slight procedural modifications. By applying an ML algorithm based on image inputs of bacterial growth pattern on SBA and the patient’s platelet count on day 1 of bacteremia, the microbiology lab would be able to provide clinicians with a risk probability for whether a patient would develop thrombocytopenia on day 4 of bacteremia onset. This first model was shown to predict the development of thrombocytopenia on day 4 with a high AUC of 0.920. We also developed a second model to predict patient mortality based only on image inputs and day 1 patient platelet count, which yielded a modest performance with an AUC of 0.711.

Hla has been previously shown to be a key virulence factor contributing to the severity of disease [7,10,11,13]. Our group has shown a significant association between MRSA strains with high Hla activity and risk of thrombocytopenia and death in patients with SAB [11]. Others have shown the feasibility of manually interpreting SA beta-hemolytic growth on SBA in predicting the risk for development of ventilator-associated pneumonia [15]. Our study extends the approach of phenotypic characterization of strain virulence to bacteremia and provides an applicable framework for clinical implementation. By incorporating ML methods to analyze the images of SA beta-hemolytic growth on SBA plates, inherent inter-operator variability from visual inspection is eliminated during interpretation, providing more robust and consistent results. In addition, neural network architecture allows for iterative improvement in image interpretation as the number of images increases over time with usage.

Our models utilize an early patient clinical variable (i.e., platelet count on day 1 of bacteremia) that represents a host response specific to SA virulence (Hla) that is already routinely measured. Thus, clinicians do not need to order specialized testing for patients to utilize our models. Further, since SA growth on SBA plates is already performed as part of routine workflow in clinical microbiology, lab technologists do not need to undergo extensive training on novel techniques or perform extra testing for data collection. Together, utilizing images of bacterial growth on SBA and day 1 platelet counts, we have shown the potential application of machine learning to develop clinically relevant prediction models that could be used to guide treatment selection directed at the host–microbe interface. We also employed inter-run validation and transfer learning methods to enhance the models’ robustness. Further validation of our models to address regulatory requirements for clinical microbiology reporting and test interpretation working collaboratively between microbiology and the antimicrobial stewardship team will also be required prior to clinical implementation.

Our study has several limitations. First, our sample size is limited for ML-based algorithm development based on image analysis. Nonetheless, we were able to demonstrate high performance metrics for predicting the development of thrombocytopenia on day 4 of infection. In addition, our proof-of-concept models were built based on the specific interaction between one virulence factor (Hla) and one host cell type (platelet). It is possible that inclusion of other relevant clinical variables may increase the performance metrics of our models. Further, SA produces other less prominent hemolysins (i.e., β-, γ-, and δ-hemolysin) which can lyse sheep blood on agar, albeit to a more limited extent [25]. While β-hemolysin has been shown to exhibit strong hemolysis of sheep blood, it is only observed following incubation of SA cultures on SBA plates at 4 °C, and thus is unlikely to contribute meaningfully to the zone of hemolysis observed under our experimental condition [26]. The other hemolysins, however, may be the source of the small zones of hemolysis seen with the few isolates that did not exhibit activity in the rabbit erythrocyte hemolysis assay.

The use of platelet-directed therapeutics (i.e., ticagrelor) in the treatment of SAB patients complicated by thrombocytopenia, has garnered interest recently in improving treatment success through mitigation of Hla-mediated platelet depletion [24,27,28]. These P2Y12 antagonists are suggested to reduce Hla-mediated platelet desialylation and subsequent clearance through the hepatic Ashwell-Morell receptor pathway, thereby allowing for platelets to remain in circulation to exert innate immune effector function. Interestingly, one study suggested that P2Y12 receptor antagonists may abrogate platelet antistaphylococcal responses by inhibiting P2Y12-mediated platelet activation and subsequent release of platelet microbicidal proteins (PMPs) [29]. Another study illustrated in a rabbit model of infective endocarditis a paradoxical association of decreased virulence potential by hyperproducing Hla strains of SA [30]. The authors suggested that excess Hla production in the local cardiac SA vegetations may result in an increased release of PMPs (secondary to either increased platelet secretion or lysis) leading to reduced virulence and greater bacterial clearance. Thus, the use of anti-platelet agents as adjunct treatment of SAB raises potential concern for interfering with platelet immune effector function. However, more recent studies have shown that P2Y12 receptor antagonists such as ticagrelor but not clopidogrel, mitigate Hla-mediated toxic effects on platelets while preserving platelet immune effector function in experimental murine models and in human [24,28,29]. Specifically, ticagrelor was shown to interfere with the ability of Hla to initiate platelet aggregation by inhibiting Hla production while not preventing platelet-bacteria bridging by soluble plasma proteins and subsequent platelet aggregation [31]. Moreover, the study identified that ticagrelor treatment prevented the formation of vegetation in a murine model of SA infective endocarditis. Additionally, others have shown that pretreatment of platelets with ticagrelor enhanced human platelet killing of SA in vitro [24,27,31]. Taken together, the above findings highlight the complexity of the host–microbe interactions at play that determine the outcome of staphylococcal infections, and in the context of this study, provide further support for applying our ML approach to guide the precision management of SAB.

In summary, this study demonstrates the feasibility of ML models for digital microbiology implementation in the clinical setting to provide prediction of disease outcome in SAB. A key advantage of our model is the use of readily available information without the need for extra diagnostic testing or personnel training. Specifically, our models utilize data related to the virulence of the infecting SA strain alongside patient clinical variables to better encompass the host–microbe relationship in driving patient outcome. Our findings highlight the potential of incorporating virulence measures into clinical decision support systems to help clinicians individualize treatment selection. Validation of our models is needed by including a larger number of images of bacterial growth pattern and clinical variables. Prospective studies employing this ML algorithm in the clinical management of SAB should be performed to confirm its benefits in improving patient outcome. 

## 4. Materials and Methods

### 4.1. Patient Cohort and Bacterial Isolate Selection

All bacterial isolates and clinical data were collected as part of a large prospective observational study of adult patients hospitalized for SAB from two affiliated medical centers in Los Angeles, CA, USA, with IRB approval; informed consent was waived. A total of 229 non-duplicate bloodstream isolates from unique patients with first episode of bacteremia were selected based on beta-hemolysis and availability of patient platelet counts on day 1 and day 4 of infection. Thrombocytopenia was defined as platelet count < 150 × 10^9^/L at time of SAB onset. Severity of illness was defined by sepsis (on first day of positive blood culture, at least 2 of the following: T > 38.3 or <36; HR > 90; WBC > 12 or <4 or >10% bands; RR > 20), severe sepsis (meets sepsis criteria plus at least one of the following: SBP < 90 or SBP drop ≥ 40 of normal, lactate > 2, Scr > 2, platelets < 100,000, Tbili > 2 or INR > 1.5, acute lung injury), and septic shock (meets sepsis criteria plus need for vasopressor therapy). Patients’ medical records were reviewed to extract relevant laboratory and clinical data and recorded using REDCap electronic data capture tools hosted at our university [32].

### 4.2. Bacterial Growth Conditions

Bacterial isolates were identified by colony morphology and use of Staphaurex™ latex agglutination test (Thermo Fisher, Waltham, MA, USA). Isolates stored in frozen glycerol stocks were sub-cultured; then, three representative colonies of each study isolate were inoculated into 5 mL Tryptic Soy Broth (TSB) and incubated overnight at 37 °C in ambient air with shaking at 250 rpm. The liquid cultures were then streaked onto TSA containing 5% sheep blood (SBA) (Hardy Diagnostics, Santa Maria, CA, USA) and incubated at 37 °C in ambient air for 20 h. For each isolate, a minimum of two replicates of plated bacterial growth were used to generate the images for the development of the machine learning algorithm. A total of 539 images of bacterial growth on sheep blood agar were generated from 229 patients with at least two replicate images generated from each patient.

### 4.3. Rabbit Erythrocyte Lysis Assay

To profile Hla phenotype of study isolates, bacterial supernatants were generated and underwent sterile filtration then stored at −80 °C until later analysis as we have done previously [11]. All cell-free supernatants were normalized to 1 × 10^9^ CFU/mL for the hemolysis assay using rabbit erythrocytes (Innovative research, Novi, MI, USA) to measure the hemolytic activity of Hla, defined as the inverse of the dilution causing 50% hemolysis (in hemolytic units per mL, HU/mL). All supernatants were tested in duplicates in two independent experiments and the results were averaged.

### 4.4. Machine Learning Algorithm Development

A smartphone (Galaxy Fold 3, Samsung, South Korea) was used by a single operator to take one image per plated bacterial growth on SBA for each bloodstream *S. aureus* isolate. For the isolates displaying visible zones of beta-hemolysis, masks were generated using the LabelMe program and the OpenCV Python library, then cropped to remove extraneous data [33,34]. The images were then resized to 350 × 350 pixels. The pixel values were then normalized to be in the range of 0–1 rather than 0–255 to ensure similarity in the distribution of the pixel values across images, aiding convergence of the machine learning models. Associated with each image are day 1 and day 4 platelet counts of the patient from which the isolate was obtained, and 30-day mortality.

The target variables in the two models developed are thrombocytopenia at day 4 (platelet counts < 150 × 10^9^/L) and mortality. In total, 36.4% of the images employed were from patients who developed thrombocytopenia and 63.6% were from patients who did not. A total of 78.1% of images came from patients who survived, while 21.9% came from patients who did not. Due to imbalanced groups in the dataset, the training data for the mortality prediction model employed upsampling on the minority class, bringing the distribution to 63.8% in the “survivor” category and 36.2% in the “non-survivor” category. This upsampling was only performed on the training data and not on the testing data. The neural network architectures employed in all experiments are InceptionV3 and ResNet50 [35,36]. Pretraining with the ImageNet database was utilized to improve model performance given the relatively low number of plate images available for this study. The pretrained convolutional layers were followed by a global average pooling layer employed to the convolved images into 1-dimensional vectors, then followed by a dense layer with 256 neurons and a dropout rate of 0.2 (included to combat overfitting). The output of this dense layer was concatenated with the patient’s day 1 platelet count, and this vector was then fed into a final dense layer.

Using images of bacterial growth on SBA and the day 1 patient platelet count as inputs, two models were constructed to predict (1) thrombocytopenia at day 4 (platelet counts < 150 × 10^9^/L) and (2) mortality. Experiments to predict the occurrence of thrombocytopenia (model 1) and 30-day patient mortality (model 2) were conducted using stratified 5-fold cross validation. Average receiver operating characteristic (ROC) curves were generated for each model to determine their aggregate performance as measured by the area under the curve (AUC).

## Figures and Tables

**Table 1:** Patient demographics, clinical presentation, and outcome.

	All Patients (n = 229)	Normal Platelet (n = 151)	D1 Thrombocytopenia (n = 78)	*p* Value
Age, y, mean (SD)	60 (16.4)	60 (16.6)	60 (16.0)	0.96
Sex (male)	163 (71.2)	111 (73.5)	52 (66.7)	0.29
Comorbidities				
None	24 (10.5)	14 (9.3)	10 (12.8)	0.50
Coronary artery disease	30 (13.1)	16 (10.6)	14 (17.9)	0.15
Heart failure	30 (13.1)	19 (12.6)	11 (14.1)	0.69
Diabetes	98 (42.8)	65 (43.0)	33 (42.3)	>0.99
Hypertension	116 (50.7)	82 (54.3)	34 (43.6)	0.13
Intravenous drug use	35 (15.3)	23 (15.2)	12 (15.4)	>0.99
Liver disease	41 (18.0)	22 (14.6)	19 (24.4)	0.072
Renal disease	65 (28.4)	41 (27.2)	24 (30.8)	0.64
Dialysis	47 (20.5)	29 (19.2)	18 (23.1)	0.49
Source risk category				
Low ^a^	49 (21.4)	33 (21.9)	16 (20.5)	0.87
Intermediate ^b^	129 (56.3)	94 (62.2)	35 (44.9)	0.017
High ^c^	51 (22.3)	24 (15.9)	27 (34.6)	0.0023
Microbiology				
MRSA	84 (36.7)	53 (35.1)	31 (39.7)	0.56
Severity of illness				
Sepsis	197 (86.0)	126 (83.4)	71 (91.0)	0.16
Severe sepsis	122 (53.3)	63 (41.7)	59 (75.6)	<0.0001
Septic shock	35 (15.3)	15 (9.9)	20 (25.6)	0.0032
Concurrent antiplatelets	61 (26.6)	39 (25.8)	22 (28.2)	0.75
Duration of SAB, days	3 (1–5)	2 (1–4)	4 (1–6)	0.0016
Persistent SAB	91 (39.7)	48 (31.8)	43 (55.1)	0.001
30-day mortality	43 (18.8)	17 (11.3)	26 (33.3)	0.0001

Note: ^a^ osteoarticular, soft tissue, and unknown sources; ^b^ intravenous catheter, urinary tract infection, ear-nose-larynx, gynecologic, digestive endoscopy, arterial catheterization, and sclerosis of esophageal varices; ^c^ endovascular sources, lower respiratory tract, intra-abdominal, and central nervous system foci. Infection source risk categories are defined by related mortality rate to determine low (<10%), medium (10–20%), and high (>20%) groups [22]. Data are n (%) or median (IQR). Persistent SAB is defined by greater than or equal to 4 days of positive blood cultures. Severity of illness is defined by sepsis (on first day of positive blood culture, at least 2 of the following: T > 38.3 or <36; HR > 90; WBC > 12 or <4 or >10% bands; RR > 20), severe sepsis (meets sepsis criteria plus at least one of the following: SBP < 90 or SBP drop ≥ 40 of normal, lactate > 2, Scr > 2, platelets < 100,000, Tbili > 2 or INR > 1.5, acute lung injury), and septic shock (meets sepsis criteria plus need for vasopressor therapy). *p* ≤ 0.05 denotes statistical significance as determined by Student’s t-test, Fisher’s exact test, or Mann–Whitney test where appropriate.

**Table 2:** Performance metrics for ML-based models from 5-fold cross-validation experiments.

Metric	Model 1: Day 4 Platelet Count		Model 2: Mortality	
	Mean	Standard Deviation	Mean	Standard Deviation
Accuracy	0.823	0.032	0.691	0.064
F1-Score	0.806	0.049	0.511	0.025
Precision	0.782	0.102	0.392	0.045
Sensitivity	0.891	0.082	0.750	0.076
Specificity	0.724	0.187	0.672	0.101
AUC	0.920	0.027	0.711	0.048

Note: Accuracy: number of correct predictions among all predictions. Precision: number of correctly identified positive predictions. Sensitivity: proportion of correct positive predictions among all positive predictions. Specificity: proportion of correct negative predictions among all negative predictions. F1-Score: combined average of precision and sensitivity. Area under the Curve (AUC): aggregate performance metric across classification thresholds.

## Data Availability

The datasets used and/or analyzed during this study are available from the corresponding author upon reasonable request.

## Supplementary Materials

The following supporting information can be downloaded at: https://www.mdpi.com/article/10.3390/toxins15070417/s1, Table S1: Study strain hemolytic activity data.
